# Treatment of Silk Fibroin with Poly(ethylene glycol) for the Enhancement of Corneal Epithelial Cell Growth

**DOI:** 10.3390/jfb6020345

**Published:** 2015-05-29

**Authors:** Shuko Suzuki, Rebecca A. Dawson, Traian V. Chirila, Audra M. A. Shadforth, Thomas A. Hogerheyde, Grant A. Edwards, Damien G. Harkin

**Affiliations:** 1 Queensland Eye Institute, South Brisbane, Queensland 4101, Australia; E-Mails: shuko.suzuki@qei.org.au (S.S.); audra.shadforth@qei.org.au (A.M.A.S.); thomas.hogerheyde@qei.org.au (T.A.H.); 2 School of Biomedical Sciences, Faculty of Health, Queensland University of Technology, Brisbane, Queensland 4001, Australia; E-Mails: r2.dawson@qut.edu.au (R.A.D.); d.harkin@qut.edu.au (D.G.H.); 3 Science and Engineering Faculty, Queensland University of Technology, Brisbane, Queensland 4001, Australia; 4 Faculty of Medicine and Biomedical Sciences, University of Queensland, Herston, Queensland 4029, Australia; 5 Australian Institute for Bioengineering and Nanotechnology, University of Queensland, St Lucia, Queensland 4072, Australia; E-Mail: g.edwards1@uq.edu.au; 6 Faculty of Science, University of Western Australia, Crawley, Western Australia 6009, Australia; 7 Institute of Health and Biomedical Innovation, Queensland University of Technology, Kelvin Grove, Queensland 4059, Australia

**Keywords:** silk, *Bombyx mori* silk fibroin, membranes, poly(ethylene glycol), porosity, surface topography, permeability, corneal epithelial cells, cell attachment

## Abstract

A silk protein, fibroin, was isolated from the cocoons of the domesticated silkworm (*Bombyx mori*) and cast into membranes to serve as freestanding templates for tissue-engineered corneal cell constructs to be used in ocular surface reconstruction. In this study, we sought to enhance the attachment and proliferation of corneal epithelial cells by increasing the permeability of the fibroin membranes and the topographic roughness of their surface. By mixing the fibroin solution with poly(ethylene glycol) (PEG) of molecular weight 300 Da, membranes were produced with increased permeability and with topographic patterns generated on their surface. In order to enhance their mechanical stability, some PEG-treated membranes were also crosslinked with genipin. The resulting membranes were thoroughly characterized and compared to the non-treated membranes. The PEG-treated membranes were similar in tensile strength to the non-treated ones, but their elastic modulus was higher and elongation lower, indicating enhanced rigidity. The crosslinking with genipin did not induce a significant improvement in mechanical properties. In cultures of a human-derived corneal epithelial cell line (HCE-T), the PEG treatment of the substratum did not improve the attachment of cells and it enhanced only slightly the cell proliferation in the longer term. Likewise, primary cultures of human limbal epithelial cells grew equally well on both non-treated and PEG-treated membranes, and the stratification of cultures was consistently improved in the presence of an underlying culture of irradiated 3T3 feeder cells, irrespectively of PEG-treatment. Nevertheless, the cultures grown on the PEG-treated membranes in the presence of feeder cells did display a higher nuclear-to-cytoplasmic ratio suggesting a more proliferative phenotype. We concluded that while the treatment with PEG had a significant effect on some structural properties of the *B. mori* silk fibroin (BMSF) membranes, there were minimal gains in the performance of these materials as a substratum for corneal epithelial cell growth. The reduced mechanical stability of freestanding PEG-treated membranes makes them a less viable choice than the non-treated membranes.

## 1. Introduction

The silk produced by the larvae of domesticated silkmoth (*Bombyx mori*) or some wild silkmoths have been known in the textile manufacturing for millennia [1,2,3,4]. In medicine, the use of silk threads as surgical sutures can be traced back to the beginning of the Common Era, when it was suggested by Galen of Pergamon [5,6]. With the increasing availability of *B. mori* silk throughout the subsequent centuries, the silk sutures became steadily used and, starting with the 19th century [7], they dominated the surgical field owing to some remarkable properties [8,9,10,11]. In 1866, Williams used for the first time silk sutures in the eye surgery in cataract operations [12], and Kuhnt followed his example in corneoscleral surgery [13]. Relatively slowly, silk became the suture material of choice in ophthalmic surgery [14,15,16,17]. Today, although the silk sutures are still available on the market and in clinical use, the sutures made of synthetic polymers (such as polyamides, polyesters, lactone-based polymers, and polyolefins) are generally preferred by surgeons. However, the medical applications of *B. mori* silk have not stopped at sutures. With the significant progress over the last few decades in understanding the complex structure and composition of silk and with the advent of methods enabling the isolation of its polypeptidic components, new applications emerged for the two main constitutive proteins of silk, fibroin and sericin [18,19,20]. Due to an array of desirable properties (they can be processed into various forms; do not elicit toxic or traumatic effects to living tissues; elicit low immune response; are permeable for oxygen, fluids and biomolecules; degrade protractedly in physiologic media and the resulting products do not accumulate in the body; and fibroin, in particular, also displays suitable mechanical strength), the silk proteins have been extensively investigated as biomaterials for tissue engineering, regenerative medicine and sustained drug delivery [21,22,23,24,25,26,27,28,29,30,31,32,33].

The feasibility of utilizing silk proteins as biomaterials for reconstructing tissue of clinical significance in the human eye was first reported by our group when we demonstrated that primary human corneal limbal epithelial cells could attach and proliferate on membranes of *B. mori* silk fibroin (BMSF) at levels comparable to those observed on tissue culture plastic (TCP) substrata, both in serum-supplemented and serum-free media [34,35]. Subsequent work has established BMSF as a functional substratum of significant potential in ocular tissue engineering [36,37,38,39]. Our investigations extended also to *B. mori* sericin [40], and to the fibroin produced by a wild species of silkmoth, *Antheraea pernyi* [41,42]. We have reported extensively on the evaluation of silk proteins as substrata for corneal cells (epithelial, limbal epithelial, limbal mesenchymal stromal, endothelial) [34,35,36,40,41,42,43,44,45,46], and retinal pigment epithelial cells [37,47].

For ocular tissue-engineered constructs, the templates should ideally be thin (2–10 μm), transparent, flexible, strong enough for surgical manipulation, permeable to solutes, and should promote adequate levels of cell attachment and growth. While most of these prerequisites are fulfilled by the membranes made of BMSF, there is still a need to optimize some properties. Indeed, it can be said that the attachment of cells to BMSF substrata is generally weak when compared to other materials. The enhancement of substratum’s transport properties and of the adhesion and growth of cells would be important for the development of better tissue-engineered constructs, and strategies to achieve it have been actively pursued by some dedicated research groups. To this aim, methods for creating surface topographic features and/or rendering the substratum porous were investigated in order to improve colonization by corneal cells of the BMSF templates. One of strategies consists of mixing poly(ethylene glycol) (PEG), a water-soluble polymer, into the solutions of BMSF prior to stabilizing the structure by conversion to the conformation “Silk II” that makes the membrane insoluble in water. Subsequent washing in water removes PEG, which thus fulfills its role as a porogen. NOTE: The nomenclature for PEG needs, perhaps, some clarification. Poly(ethylene oxide) (PEO) is frequently used as an alternative name, usually when the molecular weight (MW) of the polymer is over 20 kDa, although this is rather a non-abiding convention. Equivalent names, such as “polyoxyethylene” or “polyoxirane”, are seldom used, while the official IUPAC-recommended name, “poly(oxyethane-1,2-diyl)”, is never seen in literature. In this report, we will use exclusively the acronym PEG regardless of MW.

The first use of PEG to modify the properties of BMSF, with the explicit aim of generating porosity, has been reported by Asakura and coworkers [48,49]. Their objectives have been either to study the interaction between metal ions trapped within the porous structure of BMSF [48] or to enhance the permeability of the BMSF membranes used for enzyme immobilization [49]. PEG with a MW of 300 Da was used, which probably explains why no microscopic evidence for pores could be obtained, as the size (more precisely the diameter of an equivalent sphere) of this particular PEG molecule is only about 1 nm [50]. However, the roughness of the membrane surface and the permeability of membranes were both enhanced significantly as the weight ratio PEG/BMSF increased. For instance, at a weight ratio PEG/BMSF of 3, the permeability to glucose or to salt increased 20 times. As a drawback, the mechanical strength and elasticity were drastically reduced with increasing PEG content [49]. Nevertheless, Asakura’s studies have revealed that the incorporation of PEGs, at least of those with low MWs, into BMSF led not only to an increase of the permeability but also to changes in the surface topography.

Following the recognition of BMSF as a potential biomaterial, its blending with relatively low amounts of PEG with a much higher MW (900 kDa, which corresponds to a molecular size in the region of 100 nm [50]) has been investigated as a method either to reduce the brittleness of BMSF templates (as fibrous scaffolds or membranes) [51,52], or to induce porosity [53]. PEG blending also served in fundamental studies to create a model mimicking the behavior of natural silk proteins *in vivo* [54]. In the field of ocular tissue engineering, PEG with a MW of 900 kDa has been used to induce porosity in the BMSF membranes as substrata for corneal cells [44,55] or retinal cells [47], while PEG with a MW of 300 Da has been used with the same aim of improving the growth of corneal epithelial cells [56], the latter study being in fact a continuation of Asakura’s work applied in ophthalmic tissue engineering. The effects upon corneal cells’ growth of differing surface topographic patterns, created by lithographic techniques on the surface of BMSF membranes, have been also investigated on both porous [55] and non-porous membranes [57].

By using a PEG with high MW (900 kDa) as a porogen, well defined and microscopically detectable porous features were achieved in the BMSF membranes, but their performance as substrata for corneal cells was inferior to that of non-porous membranes [44,47,55]. The use of a PEG with a much lower MW (300 Da = 0.3 kDa) led to BMSF films (coated on cell culture inserts that are porous) with increased permeability and roughness of the surface [56]. While the rough topography was evident under the microscope, it appears that no pores could be seen inside the material. Remarkably, the cultures of primary rabbit corneal limbal epithelial cells on the PEG-treated substrata resulted in stratified epithelial layers, while only monolayers were noticed on the original BMSF substrata [56]. This finding could be indeed a consequence of favorable combined effects of higher permeability and rougher surface topography. The use of an underlying layer of 3T3 murine fibroblasts as feeder cells in this study almost certainly contributed to the improved growth of cells of the BMSF membranes with higher permeability. Nevertheless, the authors did not compare the growth in the presence and absence of the feeder cells. The precise mechanism of PEG action remains therefore somewhat unclear.

In the present report, we compared the attachment and proliferation of human corneal epithelial cells (HCECs as a cell line) and of human corneal limbal epithelial cells (HCLECs) on BMSF membranes that either were treated with PEG (MW 300 Da) or were not treated. Although the processing of substrata was similar to that described by Higa *et al.* [56], our study was different in many respects, including: human-derived cells instead of animal cells; freestanding BMSF membranes instead of porous culture membranes coated with BMSF films; and crosslinked membranes for enhanced mechanical stability. Moreover, we compared the growth of primary cell cultures both in the presence and absence of the feeder cells. Other differences will be discussed in the next section of this report. The aim of this study was to investigate whether the treatment of BMSF substrata with a PEG of low MW is of benefit to corneal epithelial cellular growth due to the potential synergism of higher permeability and irregular patterning of the surface.

## 2. Results and Discussion

### 2.1. Background

Being associated inherently with an enhancement of permeability, the presence of pores in the templates for cellular constructs is beneficial for the cells’ growth due to increased diffusion of oxygen, nutrients and biomolecules that must be supplied to the cells and regenerating tissue, and to improved diffusion-based waste transport. Porosity also has favorable effects on the intercellular communication and signaling, and on the spatiotemporal control of the regions where the cells are expected to operate [58,59]. Validity of these general principles for the system BMSF/ocular cells (corneal or retinal) has been investigated in some studies [44,47,55,56]. It has been found [55] that immortalized human corneal stromal fibroblasts were able to colonize stacked BMSF layers (each 2 μm thick), where pores of size from 0.5 to 5 μm were created by treatment with PEG (900 kDa), but no comparative quantitative evaluation of cellular growth was provided. Our group has previously reported [44] BMSF membranes (thickness 2.3 ± 1 μm), where pores (2.9 ± 1.5 μm) were made by the use of the same PEG (900 kDa), which were evaluated *in vitro* as substrata for cultures of human corneal limbal epithelial cells (HCLECs). The relatively larger number of cells attached on the porous BMSF as compared to non-porous BMSF substrata or TCP was not statistically significant. On the non-porous substratum, cultivation of HCLECs for two weeks resulted in stratified layers of cells with a basal cuboidal layer. In contrast, cells on the porous substratum formed flattened and squamous monolayers. The same porous BMSF membranes have also been used as substrata for the growth of retinal pigment epithelial (RPE) cells (line ARPE-19) [47]. It was found that the attachment of cells was inferior to that on TCP, but no experimental comparison was carried out against a non-porous BMSF substratum. Based on the above results, porous morphologies induced by using a PEG of high MW appear to offer no advantages for cell growth, perhaps due to the large size of the pores (see further).

The ability of corneal cells to respond to the topography of the template has been demonstrated on a variety of materials and involving a range of topographic features. For instance, employing bovine corneal epithelial tissue explants or primary corneal epithelial cells, has shown [60,61,62] that both tissue outgrowth and cell proliferation were strongly affected by the size and number of the surface pores. These studies have been carried out on various commercially available membranes such as polycarbonate, cellulose, or polyester (Mylar^®^), over the pore size range 0.1 to 3 μm. Continuous cell layers were seen on the surfaces with the smallest pore size. At pore sizes over 0.9 μm the outgrowth and proliferation were almost halted. Comparing the growth on the same material (polycarbonate), regular hemidesmosomal adhesive structures occurred only on the surface with pores of 0.1 μm, while at higher pore size these structures were restricted, and they did not occur at all at the highest pore size or on the smooth surface. In a series of reports [63,64,65,66,67], surface topographic patterns consisting of features such as grooves and ridges were created on the surface of silicon wafers (by lithography) or polyurethane membranes (by moulding) with a pitch range between 400 and 4000 nm, the pitch being the distance between the centres of two consecutive holes. The levels of adhesion and proliferation of primary human corneal epithelial cells (HCECs) [63,67] or SV40-immortalized HCECs [64,65,66,67] were systematically investigated. While on the substrata with smooth surfaces the cells were mostly round, on the patterned surfaces they were elongated and tending to adopt a stellate morphology, as well as aligned along the grooves and ridges. Following normal incubation, the cells proliferated better on silicon wafers when the features had high pitch values, and also on the smooth surface. On the contrary, when the cells were exposed to shear stress in a laminar flow chamber, the features with lowest pitch value induced the highest level of adherent cells; at the highest pitch, the effect of topography was lost. On the patterned polyurethane substrata, however, the proliferation of both types of cells decreased as the dimensions of topographic features became smaller [67].

In a study involving BMSF [55], rabbit corneal stromal fibroblasts and immortalized human corneal stromal fibroblasts were seeded on membranes patterned with concentric circular or linear grooves. While the alignment of cells during growth was evident on the patterned surfaces, the amount of adherent cells was lower than on the smooth BMSF or TCP surfaces. In a more recent study from the same laboratory [56], the initial attachment of an immortalized HCLEC line on BMSF substrata patterned with linear grooves was greater than that on glass, smooth BMSF or BMSF surfaces with circular grooves. After eight days of culture, the situation reversed and the glass and smooth BMSF substrata supported the highest levels of cellular growth. Significant improvement in the attachment and proliferation of pig vascular endothelial cells has been reported on fibrous BMSF substrata fabricated by electrospinning [68]. However, it is problematic to ascertain whether this result is due to porosity, to surface topography, or to their combined effect.

The findings in all these studies, sometimes contradictory or difficult to interpret, illustrate the complexity of the mechanochemical signalling mechanisms governing the response of corneal cells to surface topographic cues. Notwithstanding such complexity, there might be a distinct possibility of harnessing the cells’ response for the purpose of enhancing the biocompatibility of the cell/template systems, resulting in more extensive cellular colonization of the BMSF templates and, ultimately, to functional and stable constructs for the restoration of ocular surface.

Considering the rather ambiguous results reported with a PEG of high MW [44,47,55], and the promising results reported [56] using a PEG of low MW, we developed freestanding BMSF membranes that were modified with PEG of MW 300 Da, with the expectation of increasing permeability and also of generating topographic features on the surface of the membranes. However, our approach was somewhat different from that adopted in the mentioned report [56]. Table 1 presents the experimental differences between the two studies. Critically, our studies were performed using freestanding membranes (as the substrata for clinical applications would be required), and growth of primary cultures was compared in the presence and absence of feeder cells.

**Table 1:** Comparison between experimental designs: reference [56] *vs.* this report.

Aspect	Reference [56]	This report
Cells	Primary rabbit CLECs	Primary human CLECs; SV40-immortalized HCECs
Feeder cells	Always present in cultures	Growth of primary cultures compared in the presence and absence of feeder cells
Maximum duration of cultures	7 days	12 days
Substrata	BMSF films coated onto porous cell culture membranes	Freestanding BMSF membranes
Control substrata	Non-treated BMSF film; AM	Non-treated BMSF membrane; TCP
Ratio PEG/BMSF (by wt.)	0 to 38 (assessed); 2 (recommended)	2
Mol. wt. of molecules assessed for permeability	0.376 to 15 kDa	26–28 kDa
Modification of membranes	No	Yes (by chemical crosslinking)
*In vivo* evaluation	Yes (animals)	No

CLECs: corneal limbal epithelial cells; HCECs: human corneal epithelial cells; BMSF: *Bombyx mori* silk fibroin; AM: amniotic membrane; TCP: tissue culture plastic; PEG: poly(ethylene glycol).

### 2.2. Characterization of Silk Fibroin Membranes

**Figure 1:**
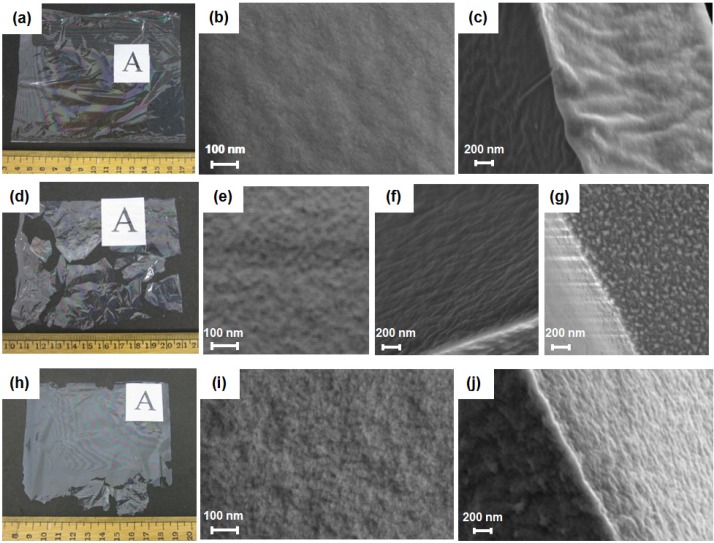
Scanning electron micrographs of the *B. mori* silk fibroin (BMSF) membranes. Physical appearance of non-treated (**a**–**c**), PEG-treated (**d**–**g**), and genipin-crosslinked PEG-treated (**h**–**j**) fibroin membranes. (**a**,**d**,**h)** Gross appearance of dried membranes after removal from the casting plate. Images of surfaces (**b**,**e**,**i**), cross-sections (**c**,**f**,**j**) and the edge of the PEG-treated membrane (**g**).

BMSF membranes of *ca.* 3 µm or *ca.* 6 µm in thickness were produced on a casting table. Upon addition of PEG with MW of 300 Da, at a PEG/fibroin weight ratio of 2, the thickness of the resulting membranes almost doubled. While the non-treated fibroin membranes were easy to peel off from the casting plate and to handle (Figure 1a), the PEG-treated membranes were fragile and difficult to remove without breaking them (Figure 1d).

To improve mechanical stability, crosslinking of PEG-treated fibroin with genipin was performed before mixing with PEG, using a previously established protocol [40]. Although the resulting membranes were thicker (10 to 15 µm), they remained more fragile than the non-treated BMSF membranes (Figure 1h). Their handling, however, became somewhat easier than of the uncrosslinked PEG-treated membranes. With care, therefore, a sufficient number of genipin-crosslinked PEG-treated membranes of suitable size could be generated for the next experiments.

Scanning electron microscopy revealed that the surfaces of PEG-treated membranes, either uncrosslinked (Figure 1e) or crosslinked (Figure 1i) were rougher than that of non-treated membranes (Figure 1b), and no pores were noticeable. These findings are in agreement with previous reports [49,56]. In cross-section, the PEG-treated membranes also showed rough morphologies (Figure 1f,j), whereas the fractured surface of the non-treated membranes was smoother and denser (Figure 1c).

**Figure 2:**
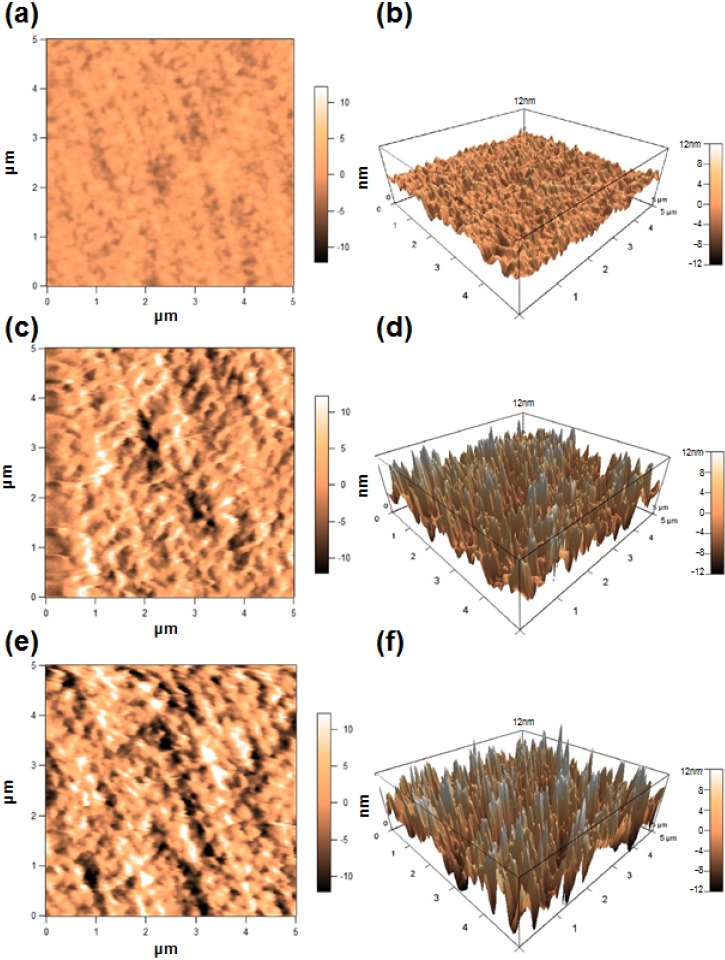
2-D and 3-D AFM images of the surfaces of non-treated (**a**,**b**), PEG-treated (**c**,**d**) and genipin-crosslinked PEG-treated (**e**,**f**) fibroin membranes. Analysed area: 5 µm × 5 µm.

In the case of the uncrosslinked PEG-treated membrane, nanoscale fibroin globules were observed mainly in a region close to the edge of the membrane (Figure 1g), which has been a general occurrence on the BMSF substrata reported previously [56]. The surface roughness of membranes was further investigated by contact mode atomic force microscopy (AFM) (Figure 2). The roughness average (*R*_a_) values measured from these images are given in Table 2. It is obvious that the treatment with PEG induced a significant increase in the value of *R*_a_, very likely due to phase separation induced through its presence.

**Table 2:** Roughness average of membranes estimated by AFM.

Fibroin membrane	*R*_a_ (nm)
Non-treated	1.3
PEG-treated	4.4
Genipin-crosslinked PEG-treated	5.9

**Figure 3:**
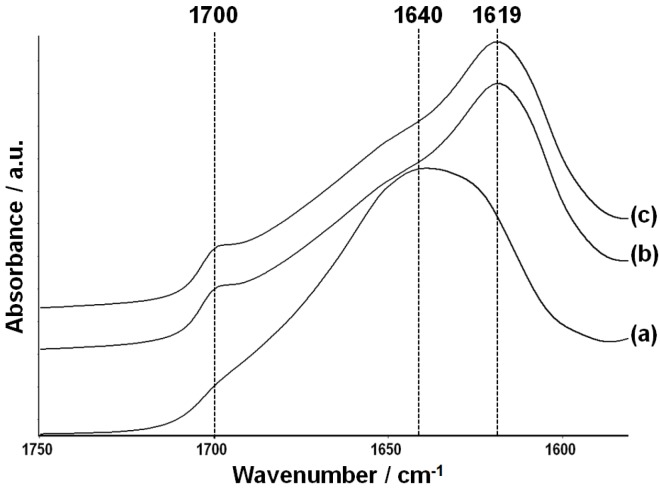
Fourier-transform infrared spectroscopy-ATR spectra of non-treated (**a**), PEG-treated (**b**), and genipin-crosslinked PEG-treated (**c**) fibroin membranes.

Infrared spectroscopy was employed to characterize the secondary structure of BMSF in the membranes [69,70]. The spectra in the Amide I region (1590–1720 cm^−1^) of the annealed non-treated membrane and of the PEG-treated membranes are shown in Figure 3. The spectrum of the non-treated membrane displayed a broad absorption band with a peak at 1640 cm^−1^, indicating a substantial amount of random-coil conformation (Figure 3a).

The broad shape of this band with a shoulder at 1619 cm^−1^ indicates a small, but significant, amount of β-sheet component in the non-treated BMSF. The Amide I band spectra of the PEG-treated membranes, either crosslinked or not, showed strong peaks at 1619 cm^−1^ and shoulders at 1700 cm^−1^, respectively, indicating a significant proportion of β-sheet conformations (Figure 3b,c) in both materials, and suggesting a negligible effect of the crosslinking upon the secondary structure of fibroin. More important here, the high content in β-sheet conformations proves that the PEG-treated membranes do not need to be water-annealed in order to induce the conformational conversion responsible for rendering the fibroin insoluble in water, as this process is accomplished due to the presence of PEG as a polar agent able to induce conversion to the β-sheet conformation more effectively than water.

**Figure 4:**
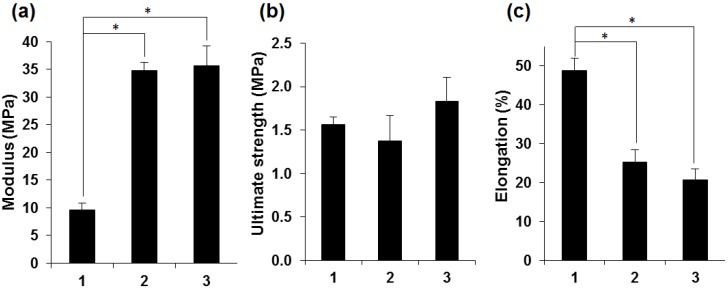
Quantitative comparison of the tensile characteristics of non-treated (1), PEG-treated (2) and genipin-crosslinked PEG-treated (3) fibroin membranes. (**a**) Young’s modulus; (**b**) Ultimate tensile strength; (**c**) Elongation at break. Bars represents mean ± standard error of the mean (*n* = 6). An asterisk indicates that the difference is statistically significant (*p* < 0.05).

The results of mechanical testing (Figure 4) indicated important differences between certain tensile characteristics of the three types of membranes. Although the ultimate strength values were similar for all samples, the elastic moduli of the PEG-treated membranes were significantly higher than those of the non-treated membranes, while their elongation at break was significantly lower. This can be a consequence of increased rigidity due to higher proportion of β-sheet conformations induced by the treatment with PEG, an assumption strongly suggested by the infrared spectrometric analysis. Rather unexpectedly, the crosslinking did not improve the tensile strength of the PEG-treated membranes.

**Figure 5:**
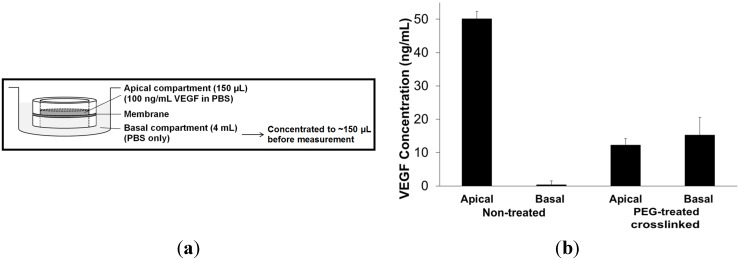
Relative permeability of BMSF membranes to VEGF. (**a**) Schematic representation of the permeability experimental setup. (**b**) Comparison of VEGF concentrations after 24 h in the apical and basal compartments delimiting the membranes.

To estimate the permeability of the BMSF membranes to biomolecules, the growth factor VEGF (vascular endothelial growth factor) was chosen as the permeant molecule, and a method was designed for the purpose (Figure 5a). VEGF has a MW of 26–28 kDa, and plays an important role in certain pathophysiological processes in the eye. In this study, we determined the relative permeability of the non-treated and of the crosslinked PEG-treated membranes. As shown in Figure 5b, the PEG-treated membranes were relatively more permeable to VEGF molecule as compared to the non-treated membranes. This clearly indicates that by blending BMSF with PEG (MW 300 Da), the permeability is enhanced, thus supporting the observations of Higa *et al*. [56]. Interestingly, approximately 50% and 70% of VEGF (*i.e.*, 7.5 and 10.5 ng) for the non-treated and PEG-treated membranes, respectively, were lost as shown by comparing the total amounts of protein in the apical and basal compartments after 24 h to the initial amounts. This could be due to the trapping of VEGF within BMSF due to electrostatic interactions of positively charged VEGF and negatively charged fibroin molecules.

### 2.3. Attachment and Proliferation of HCE-T Cell Line

**Figure 6:**
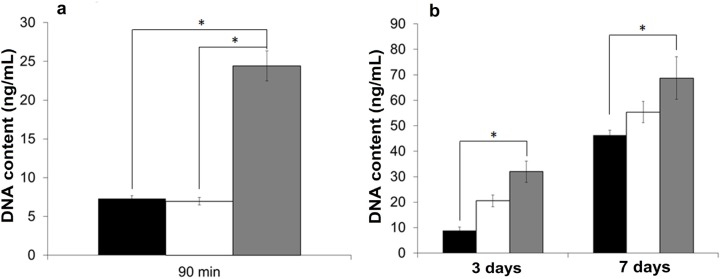
Attachment and proliferation of cells of HCE-T line on BMSF membranes. (**a**) Cellular attachment in serum-free medium; (**b**) Proliferation in serum-supplemented medium on non-treated fibroin membrane (black), genipin-crosslinked PEG-treated fibroin membrane (white) and TCP (grey). Numbers of cells were measured *via* quantification of DNA content (PicoGreen^®^ assay). Bars represent mean ± standard error of the mean. The asterisk indicates that the difference is statistically significant (*p* < 0.05).

The attachment and proliferation of an SV40-immortalized cell line (HCE-T) was examined on membranes (*ca.* 6 µm in thickness) placed at the bottom of the culture-plate wells. Since these cells can be serially propagated in the absence of feeder cells, they provided a useful model of the human corneal epithelial cells’ growth in the absence of any accessory cells. The numbers of adherent cells was expressed as the total DNA content with the PicoGreen^®^ assay (Figure 6). In a short-term attachment assay (over a period of 90 min), no quantitative difference between the numbers of cells attached to the genipin-crosslinked PEG-treated and those attached to non-treated membranes in serum-free conditions was noticed (Figure 6a), but they were significantly lower than the number of cells attached to the TCP control. In longer-term cultures (up to seven days), cell growth on the PEG-treated membrane was higher than that on non-treated membrane in serum-supplemented growth medium, albeit the differences were not statistically significant (Figure 6b). However, the level of cell growth on the non-treated membranes was found to be significantly lower than that on TCP substrata.

### 2.4. Growth of Primary Human Corneal Limbal Epithelial Cells (CLECs)

Primary cultures of human CLECs were cultivated for up to 12 days on freestanding BMSF membranes (*ca.* 6 µm) that had been mounted in Teflon^®^ cell culture chambers. The design of these chambers facilitates separation of culture medium between the upper and lower membrane surfaces. The growth of cells on genipin-crosslinked PEG-treated membranes (10 to 15 µm in thickness) was compared to that observed on non-treated membranes. Moreover, both membrane types were tested in both the presence and absence of irradiated 3T3 cells grown on the lower membrane surface.

**Figure 7:**
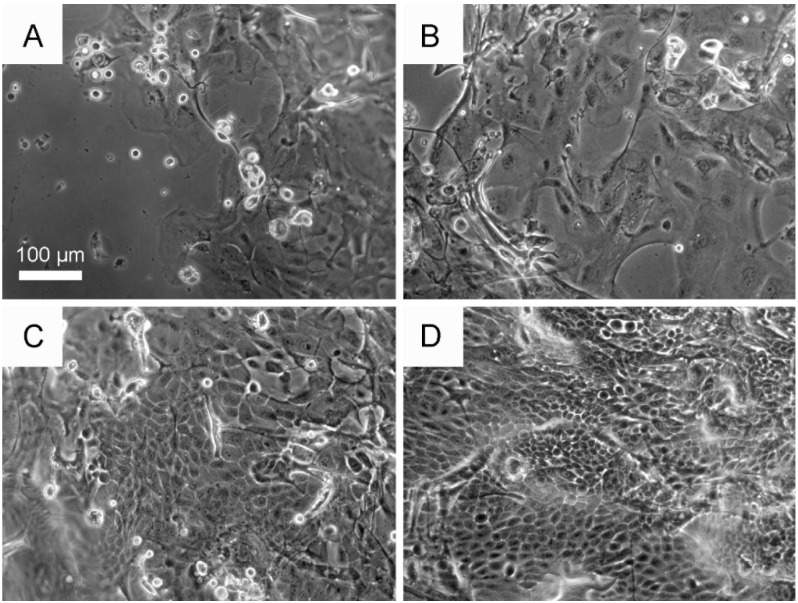
Phase contrast micrographs of primary cultures of human CLECs after five days of growth on either non-treated BMSF membranes (**A**,**C**) or genipin-crosslinked PEG-treated BMSF membranes (**B**,**D**), in either the absence (**A**,**B**) or presence (**C**,**D**) of an underlying culture of feeder cells (irradiated 3T3 murine fibroblasts).

When examined by phase contrast microscopy after five days of growth (Figure 7), a marked difference in culture morphology was observed in the presence of 3T3 cells. In short, in the presence of feeder cells, the cultures displayed a more confluent and compact morphology, which is indicative of a more proliferative phenotype, and this effect of the feeder cells was observed irrespectively of PEG-treatment.

**Figure 8:**
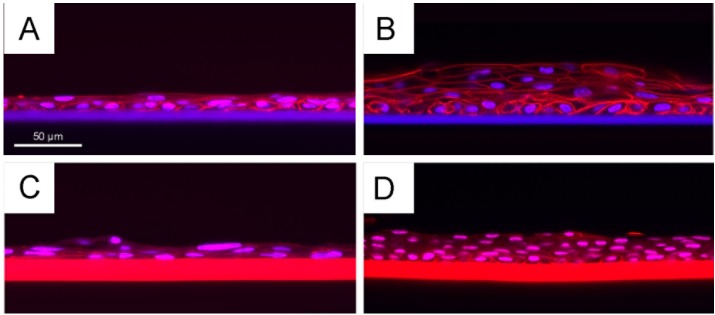
Histology by confocal microscopy after cultivation of primary human CLECs for 12 days on non-treated (**A**,**B**) and genipin-crosslinked PEG-treated (**C**,**D**) BMSF membranes: without feeder cells (**A**,**C**); co-cultured with feeder cells (irradiated 3T3 murine fibroblasts) (**B**,**D**). The feeder cells have become dislodged during culture and subsequent preparation of samples for confocal microscopy. The genipin-crosslinked PEG-treated membranes were thicker than the non-treated membranes and displayed intense auto-fluorescence, as seen in (**C**) and (**D**).

After 12 days of growth, all cultures were fixed and subsequently stained with rhodamine phalloidin (to display F-actin filaments) and Hoechst nuclear dye (to display cell nuclei). Using confocal fluorescence microscopy, a high-resolution optical cross-section was obtained through each culture when folded and mounted in glycerol under a glass coverslip (Figure 8). This technique revealed that human CLEC cultures grown on fibroin membranes are consistently more stratified when an underlying layer of irradiated 3T3 cells is present, and the stratification was observed irrespectively of treatment with PEG. Nevertheless, the cells present within the cultures grown on PEG-treated membranes, in the presence of feeder cells, displayed a higher nuclear-to-cytoplasmic ratio suggesting a more proliferative phenotype. This observation tends to support the conclusions of Higa *et al.* [56] that superior growth is seen using PEG-treated membranes. Logically, this enhanced growth is due at least in part to the presence of feeder cells, but since cultures grown on non-treated membranes also displayed increased stratification, we cannot discount the potential role of changes in membrane topography created by PEG in conjunction with effects mediated by the feeder cells.

Ultimately, both membranes may well support the manufacture of human CLEC cultures of sufficient quality to enable therapeutic applications. Nevertheless, the difficulties that we encountered in producing freestanding PEG-treated membranes suggest that any potential benefits bestowed by this material are insufficient to warrant changing our clinical strategy.

### 2.5. Summary

As this study has duplicated experiments previously reported [56], albeit with some important differences (see Table 1), we expected that our results would confirm those findings. While the PEG-treated membranes were called “porous” [56], there was no microscopic evidence for pores in the bulk of material, *i.e.*, in the cross-sectioned membranes. The only micrographs provided (see “Fig. 2A,B” in ref. [56]) were those of the membrane surfaces, which showed that the surfaces became rougher following treatment with PEG (MW 300 Da). Our study confirms this observation, and also confirmed that the permeability increased after treatment with PEG.

No difference in cell attachment and growth was observed in the experiments using a transformed human corneal epithelial cell line. However, the results obtained using primary cultures of human corneal limbal epithelial cells suggested that an enhanced permeability of PEG-treated membranes does lead to subtle changes in cell behaviour that could be of a clinical value (a more proliferative cell phenotype). Nevertheless, since an underlying culture of irradiated 3T3 murine fibroblasts (as feeder cells) can also influence positively the growth of HCECs when cultivated on non-treated membranes, it would appear that even without PEG-treatment the BMSF substrata are perhaps sufficiently permeable to support the manufacture of clinically suitable cultures. Ultimately, further studies in a pre-clinical model of ocular surface disease will be needed to resolve this issue. From our perspectives, we believe that the technical difficulties associated with the routine manufacture of freestanding PEG-treated membranes will outweigh any potential benefits arising from the apparent increase in permeability of the substratum.

A rather tenuous cell adhesion is a known drawback of the BMSF templates [71]. However, the biomaterial characteristics of fibroin make these templates attractive for tissue engineering applications, as proved by the growing number of the published studies, and the enhancement of cell attachment to BMSF remains therefore a topic of great interest. Rather than cell adhesion based on non-specific interactions, which likely govern this process on BMSF, there is a need to promote physicochemical characteristics in the substratum’s surface that will be able to mediate the cell-surface anchorage in a specific way. Ideally, the surface shall comprise structural elements leading to its recognition by the cells’ integrin receptors and thus generating true focal adhesions between cells and surface. Whether the modification of BMSF surface through covalent binding of extracellular matrix proteins or/and through topographic patterning are sufficient for introducing specific interactions in order to facilitate stronger cell attachment is yet to be determined, notwithstanding the volume of research dedicated to this topic. The contribution of higher porosity and/or permeability is limited to improving intercellular communication, however without promoting specific interactions.

## 3. Experimental Section

### 3.1. Materials

*Bombyx mori* silkworm cocoons (with pupae removed) were purchased from Tajima Shoji Co. Ltd. (Yokohama, Japan). Genipin (98% purity) was supplied by Erica Co. Ltd. (Shenzhen, China). Topas^®^ 8007S-04 (olefin copolymer) was supplied by Topas Advanced Polymers (Frankfurt, Germany). Minisart^®^-GF pre-filters (0.7 µm) and Minisart^®^ filters (0.2 µm) were supplied by Sartorius Stedim Biotech (Göttingen, Germany), and the dialysis cassettes Slid-A-Lyzer^®^ (MWCO 3.5 kDa) by Thermo Scientific (Rockford, IL, USA). Sodium carbonate, lithium bromide, PEG (MW 300 Da) and 10% formaldehyde solution were supplied by Sigma-Aldrich. High purity water (Milli-Q) was used in all experiments. The human vascular endothelial growth factor (VEGF, #3045-VE-025/CF) and its enzyme-linked immunosorbent assay (ELISA) kit (#DY3045) were purchased from R&D Systems (Melbourne, Australia). Amicon Ultra-4 centrifugal filters (#UFC801024, 10 kDa MWCO) were supplied from Merck Millipore Ltd. (Darmstadt, Germany). Foetal bovine serum (FBS) was supplied by Thermo Scientific (USA). All other cell culture reagents and supplements, as well as Quant-iT™ PicoGreen^®^ dsDNA assay kit were purchased from Life Technologies (Melbourne, Australia).

### 3.2. Preparation of Fibroin Membranes

Silk fibroin solution was prepared according to a previously established protocol [40]. The concentration of solution used in experiments was 1.78% (as determined by gravimetric analysis). The standard BMSF membranes were prepared by casting the fibroin solution in a custom-made casting table where the supporting glass plate was pre-coated with a polyolefin polymer (Topas^®^) film [45]. The blade height was set in order to generate an approximate dry thickness of either 3 µm or 6 µm for the resulting BMSF membranes. After drying, the membranes were water-annealed in a vacuum chamber at −80 kPa for 6 h at room temperature in the presence of a container filled with water, followed by peeling off from the supporting Topas^®^ film.

The PEG-treated BMSF membranes were prepared according to a published protocol [56], with some modifications. In brief, PEG was slowly blended into the 1.78-% fibroin solution at a PEG/fibroin ratio of 2 (by weight). The solution was cast as described above. After drying, the membranes were soaked in 2 L of water for 3 days with two water exchanges per day to remove PEG. The dried membrane was then peeled off from the underlying Topas^®^ film.

In order to crosslink the PEG-treated membranes, an amount of genipin equivalent to 12 wt% of fibroin was mixed with fibroin solution and stirred slowly for 5 h at 40 °C [40]. The mixture acquired a light blue hue, which is indicative of a reaction taking place between genipin and amino acids [72]. Subsequently, the membranes were processed following the method described above for the PEG-treated BMSF membrane.

### 3.3. Scanning Electron Microscopy (SEM)

Small pieces of 3-µm thick membranes or freeze-fractured fragments (the latter for the examination in cross-section), were placed on specimen stubs using double-sided adhesive tapes and coated with a layer of iridium using a sputter coater. Field-emission scanning electron microscopy (FE-SEM Sigma, Zeiss, Germany) was employed to examine the surface and internal morphologies of various membranes.

### 3.4. Atomic Force Microscopy (AFM)

Fibroin films were cast on clean glass slides. A MultiScan AFM (BMT, Ettlingen, Germany) in contact mode was employed using a silicon cantilever (ContAl-G, BudgetSensors, Bulgaria), with a tip radius of less than 10 nm and a scan rate of 0.50 Hz. The *R*_a_ value was obtained from the total area of 5.0 × 5.0 µm^2^ of the AFM image.

### 3.5. Fourier-Transform Infrared Spectroscopy (FTIR)

A Nicolet FTIR spectrometer (Thermo Electron Corp., Waltham, MA, USA), equipped with a diamond attenuated total reflectance (ATR) sampling accessory, was used to analyse the secondary structure of each type of BMSF membrane. Each spectrum was obtained by co-adding 64 scans over the range 4000 to 525 cm^−1^ at a resolution of 8 cm^−1^. The OMNIC 7 software package (Thermo Electron Corp., Waltham, MA, USA) was used to analyse and plot the spectra.

### 3.6. Tensile Testing

Strips (1 cm × 3 cm) cut out from each of the 3-µm thick membranes were subjected to tensile measurements using an Instron 5848 microtester (Instron, UK), equipped with a 5 N load cell, at a crosshead speed of 14 mm/min. The stripes were loaded by pneumatic grips, which were set to a gauge distance of 14 mm, and soaked in phosphate buffered saline (PBS) (pre-heated to 37 ± 3 °C) in a BioPuls™ unit for 5 min prior to stretching. Stress-strain plots were recorded and Young’s modulus was determined from the slope of the linear region of the curve. The mean values were calculated from six measurements of each membrane.

### 3.7. Permeability of the Membranes

A permeability test was designed to quantify the movement of biomolecules across the fibroin membranes. Custom-designed Teflon^®^ chambers (Figure 5a) were used to suspend the BMSF membrane, which creates separate upper and lower compartments. This assay uses a known concentration of vascular endothelial growth factor (VEGF) in the upper compartment, and at a set time point the movement of the VEGF molecules into the lower compartment (through the membrane) can be quantified by ELISA (enzyme-linked immunosorbent assay). VEGF is a basic protein with a MW of 26–28 kDa and an isoelectric point of 8.5.

Discs cut out of each membrane were assembled in chambers and sterilized by immersion in a 70% ethanol solution for 1 h, air-dried in a biohazard hood and rinsed 3 times with PBS. Chambers were inserted into a 6-well plate well with 4 mL fresh PBS. This volume creates the lower compartment below the membrane. A VEGF solution (100 ng/mL) was prepared and 150 µL was added to the upper compartment, above the membranes. The plate was incubated at 37 °C, and upper and lower compartment volumes were collected after 24 h. Samples were frozen at −40 °C immediately after collection. ELISA assay was performed to examine each sample volume. The purpose of the assay is to determine if a particular protein is present in a sample and, if so, how much is present. We performed the assay using a commercially available VEGF sandwich ELISA kit. Briefly, the assay TCP plates (96-well) were prepared by coating with the capture antibody, and incubated at room temperature overnight. The plates were then washed and blocked with bovine serum albumin. The upper and lower compartment volumes were thawed and added to the corresponding assay plate/wells. Lower compartment volumes were concentrated using Amicon Ultra-4 centrifugal filters (MWCO 10 kDa) to be equivalent to the upper compartment volume (about 150 µL). A VEGF standard curve was also included on each assay plate. All samples and standards were tested in duplicate. After incubation at room temperature for 2 h, each plate was washed, and the detection antibody was added, and incubated.

In order to quantify the interaction of the detection antibody, the plate was washed, and a secondary antibody coupled to horseradish peroxidase (HRP) was added and incubated for 20 min. After a final wash step, the HRP enzyme was activated using a Substrate System, incubated for 20 min, which initiated the visible colour reaction. The enzyme reaction was completed with the addition of the Stop Solution, and each plate was placed into an absorbance microplate reader. The optical density was determined for each well at 450 nm and at 540 nm. The intensity of the colour reaction is proportional to the amount of VEGF protein in the original sample volumes, *i.e.* bound to the capture antibody on the bottom of the wells.

### 3.8. Culture and Growth of Transformed Human CECs on BMSF Substrata

A SV40-immortalized cell line (HCE-T) derived from human corneal epithelial cells (CECs) was used for assaying the initial cell attachment and growth. HCE-T cells were cultured in Dulbecco’s modified Eagle medium supplemented with 10% FBS, glutamine and 1% v/v penicillin/ streptomycin. The cells were cultured in a humidified atmosphere of 5% CO_2_ at 37 °C, and passaged using Versene and TrypLE^®^.

Silk fibroin membranes were cut using a trephine blade to produce circular pieces of approximately 14 mm in diameter, and placed into individual wells of a 24-well TCP plate using rubber O-rings. They were sterilized in 70% ethanol for 30 min followed by washing three times with PBS. The HCE-T cells (20,000/cm^2^) were seeded into each well with 0.5 mL/well medium, and 0.25 mL medium was exchanged every third day. Serum-free medium was used in the case of the short-term attachment assay (90 min). For proliferation assay, the cultures were assayed after incubation for 3 or 7 days in serum-supplemented media. At the end of each time point, the O-rings were removed and the cultures were rinsed three times with PBS. The DNA content of adhered cells was quantified using the PicoGreen^®^ assay as previously described [40]. All experiments were conducted in triplicate for each series of three assessments.

### 3.9. Culture and Growth of Primary Human CLECs on BMSF Substrata

Cadaveric human eye tissue was obtained with human research ethics committee approval and donor consent from the Queensland Eye Bank, Brisbane, Australia. The primary cultures of human corneal limbal epithelial cells were established from the corneal limbus as described previously [40], and cultured in Green’s medium [73]. Freshly isolated human CLECs were seeded into 25 cm^2^ flasks containing 1 × 10^6^ irradiated 3T3 murine fibroblasts (i3T3) as feeder cells. Membranes, *ca.* 6 μm in thickness, were cut into circular pieces of approximately 14 mm in diameter and mounted in sterile Teflon^®^ cell culture chambers as described previously [43]. After sterilizing in 70% ethanol followed by rinsing with PBS, 3 × 10^4^ i3T3 cells were seeded on to the underside of the membrane and allowed to attach for 24 hours. The chambers were then inverted before 1 × 10^4^ human CLECs were seeded all in Green’s medium. The cells were cultured for 12 days with chamber re-feeds every two to three days. After 12 days, the cells were fixed by immersing the chambers in 3.7% formaldehyde and stained with rhodamine phalloidin and Hoechst nuclear dye to highlight the actin fibres and the nuclei in the cells for examination by confocal fluorescence microscopy on a Nikon A1 confocal system.

### 3.10. Statistical Analysis

The results of mechanical testing and cell culture were statistically processed by the one-way analysis of variance (ANOVA) in conjunction with Tukey-Kramer multiple comparisons, using the GraphPad Prism^®^ version 6.0.

## 4. Conclusions

The characteristics of BMSF as a substratum for the growth of corneal epithelial cells can be modified by blending with a PEG of low molecular weight such as 300 Da. Both permeability and surface topography are indeed changed in ways that are expected to be beneficial to the process of cell attachment and proliferation. In practice, however, the treatment with PEG enhances the fragility of membranes. This effect appears to negate any potential benefits to cell growth.
